# Development and Validation of a Stability-Indicating RP-HPLC Method for Duloxetine Hydrochloride in its Bulk and Tablet Dosage Form

**DOI:** 10.3797/scipharm.1009-11

**Published:** 2010-10-25

**Authors:** Usmangani K. Chhalotiya, Kashyap K. Bhatt, Dimal A. Shah, Sunil L. Baldania

**Affiliations:** Indukaka Ipcowala College of Pharmacy, Beyond GIDC Phase IV, Vithal Udyognagar, New Vallabh Vidyanagar-388121, Anand, Gujarat, India

**Keywords:** RP-HPLC, Duloxetine hydrochloride, Validation, Stress conditions, Degradants

## Abstract

The objective of the present work was to develop a stability-indicating RP-HPLC method for duloxetine hydrochloride (DUL) in the presence of its degradation products generated from forced decomposition studies. The drug substance was found to be susceptible to stress conditions of acid hydrolysis. The drug was found to be stable to dry heat, photodegradation, oxidation and basic condition attempted. Successful separation of the drug from the degradation products formed under acidic stress conditions was achieved on a Hypersil C-18 column (250 mm × 4.6 mm id, 5μm particle size) using acetonitrile: 0.01 M potassium dihydrogen phosphate buffer (pH 5.4 adjusted with orthophosphoric acid) (50:50, v/v) as the mobile phase at a flow rate of 1.0 ml/min. Quantification was achieved with photodiode array detection at 229 nm over the concentration range 1–25 μg/ml with range of recovery 99.8–101.3 % for DUL by the RP-HPLC method. Statistical analysis proved the method to be repeatable, specific, and accurate for estimation of DUL. It can be used as a stability-indicating method due to its effective separation of the drug from its degradation products,

## Introduction

**Fig. 1.**
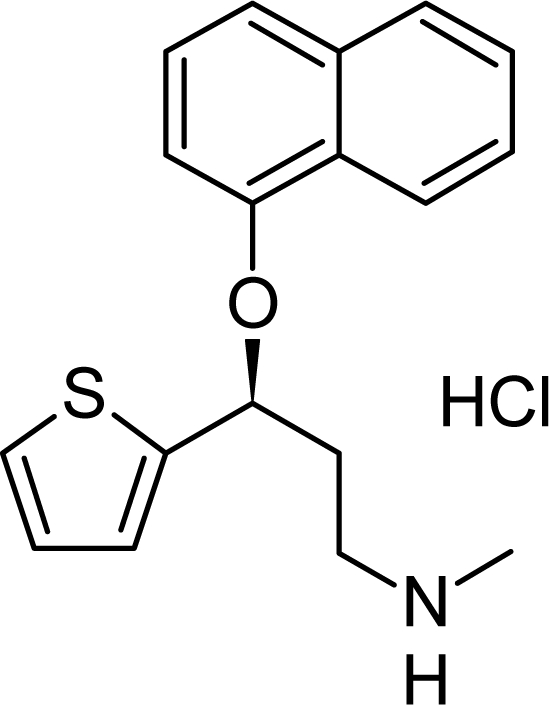
Structure of DUL

Duloxetine HCl (DUL) – (3*S*)-*N*-methyl-3-(naphthalen-1-yloxy)-3-(thiophen-2-yl)propan-1-amine hydrochloride [1] – has an empirical formula of C_18_H_19_NOS HCl and a molecular weight of 333.38 g/moL (Figure 1). It is a potent inhibitor of serotonin and norepinephrine reuptake and thus it is used for major depressive disorders [2–4]. Furthermore, it provides evidence of an effect on pain in the case of urinary incontinence [5, 6] independent of its effect on depression. Therefore, DUL is an alternative to current therapeutic options in the treatment of the different symptoms of depression [7].

A literature survey indicated few methods for the determination of DUL and its key intermediate, desmethyl-duloxetine, in human serum by HPLC [8, 9]. Reports were found regarding the characterization of phenolic impurities in DUL samples by MS, NMR spectrometry, and X-ray analysis [10] and of impurities formed by interaction of DUL with various enteric polymers [11]. A simple UV spectrophotometric method for the estimation of DUL in a formulation was reported [12]. An HPLC method separated DUL and structurally related impurities using a combination of computer-based solvent strength optimization and solvent selectivity mixture design [13]. An HPTLC method separated DUL in bulk and in tablet dosage form [14]. A capillary electrophoresis with laser-induced fluorescence detection method (Musenga et al, 2009) also reported for estimation of DUL in human plasma [15].

During our literature survey, one article related to the stability-indicating HPLC determination of DUL was found but it’s less sensitive as compare to LOD and LOQ and for linearity range is not wide [16]. International Conference on Harmonization (ICH) guidelines requires stress testing to be carried out to elucidate the inherent stability characteristics of the active drug substance [17]. The aim of the present work was to develop an accurate, selective, precise, robust, and stability-indicating RP-HPLC method for the determination of DUL in the presence of its degradation products and related impurities in tablets. The proposed method was validated according to ICH guidelines [18, 19] and its updated international convention ICH guideline on analytical method validation [20].

## Result and Discussion

### Optimization of the chromatographic conditions:

#### HPLC Method

**Fig. 2.**
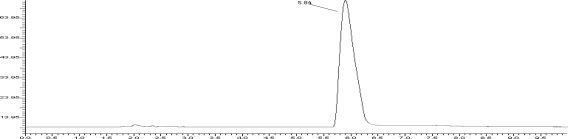
HPLC chromatogram of Duloxetine hydrochloride (RT 5.84 min) on C_18_ hypersil column using 0.01 M potassium dihydrogen phosphate buffer (pH 5.4 adjusted with orthophosphoric acid) (50:50, v/v) as the mobile phase

Several mobile phases were tried to resolve DUL but the resolution was not satisfactory. So modification was made in the above mobile phase. Finally the system containing acetonitrile: 0.01 M potassium dihydrogen phosphate buffer (pH 5.4 adjusted with orthophosphoric acid) (50:50, v/v) as the mobile phase at a flow rate of 1.0 ml/min was found to be satisfactory and gave well resolved peak for DUL. The retention time for DUL was 5.84 min. For the selection of detection wavelength, the spectrum of 10 ppm DUL revealed that, at 229 nm the drug possesses significant absorbance. So considering above fact, 229 nm was selected as a detection wavelength for estimation of DUL using HPLC. Complete resolution of the peaks with clear baseline separation was obtained (Figure 2). The system suitability test parameters are shown in Table 1.

#### Validation of the Proposed Methods

The developed method was validated, as described below, for various parameters like linearity and range, accuracy, precision, ruggedness, system suitability, specificity, LOQ, and LOD.

#### Linearity and Range

Linearity of the method was evaluated at six concentration levels by diluting the standard stock solution to give solutions in the range of 1.0–25μg/ml. The calibration curve for DUL was prepared by plotting area v/s concentration. Calibration data for DUL was shown in Table 2. The linearity plot of DUL was found to be linear with the linear equation

y=80042 x−72864

and correlation coefficient 0.998 for DUL. Linearity was observed in the expected concentration range, demonstrating suitability of the method for analysis. This indicates that the method is linear in the specified range for the analysis of DUL in dosage form.

#### Accuracy

The recovery experiments were carried out by the standard addition method. The method was found to be accurate with % recovery 99.78%–101.21% and has found with acceptable %RSD of not more than 2% at each level. The recoveries obtained by the RP-HPLC method for DUL are shown in Table 3.

#### Precision

Instrument precision was determined by performing repeatability test and the %RSD values for DUL were found to be 0.4. The intra-day and inter-day precision studies were carried out to study the precision of the developed method. For intra day study the % RSD value were found to be 0.12 – 0.36 and for interday study %RSD value were found to be 0.11 – 0.56 (Table 4). The low RSD values indicate that the method is precise.

#### Ruggedness

Ruggedness test was determined between two different days, analysts and instruments. The value of RSD was to be found 0.9 which is within acceptance criteria of below 2.0% showed ruggedness of developed HPLC method.

#### Specificity (Placebo interference)

There is no interference of mobile phase, solvent and placebo with the analyte peak and also the peak purity of analyte peak which indicate that the method is specific for the analysis of DUL in their dosage form.

#### Robustness

The method was found to be robust, as small but deliberate changes in the method parameters have no detrimental effect on the method performance as shown in Table 5. The low value of relative standard deviation was indicating that the method was robust.

#### Stability of standard and sample solutions

Stability of standard and sample solution of DUL was evaluated at room temperature for 48 hr. The relative standard deviation was found below 2.0%. It showed that both standard and sample solution were stable up to 48 hr at room temperature.

#### LOD and LOQ

These data show that the method is sensitive for the determination of DUL. The LOD and LOQ were measured by using an equation and were found to be 0.026 and 0.078 μg/ml, respectively.

#### Analysis o f a Formulation

The proposed method was applied for the determination of DUL in tablets of Duloxetine HCl. The results of these assays was 99.75% (RSD = 0.84%) of the label claim for the formulation. The results of the assay indicated that the method is selective for the assay of DUL without interference from excipients used in the tablets (Table 6).

#### Degradation Behavior of DUL

**Fig. 3.**
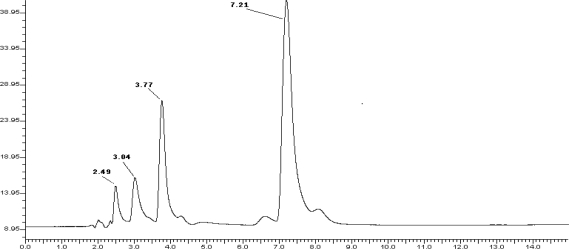
Chromatogram of acid (0.1M HCl) treated DUL at room temperature for 24 hr.

Forced degradation study was carried out by subjecting the drug to acid and alkali hydrolysis, chemical oxidation, dry heat degradation and photolytic (sun light) conditions. The chromatograms of acid degraded sample showed complete degradation product peaks at retention time (Rt) 2.49, 3.04, 3.77 and 7.21 min for DUL. The peaks of the degradation products were well resolved which is shown in (Figure 3). The DUL was found to be stable to rest of the conditions like oxidative stress degradation, dry heat degradation and alkali hydrolysis.

The degradation study thereby indicated that DUL was stable to chemical oxidation study, dry heat and alkali hydrolysis while it was highly susceptible to acid hydrolysis (Table 7).

## Conclusions

A validated stability-indicating HPLC analytical method has been developed for the determination of DUL in bulk and in tablet dosage form. The results of stress testing undertaken according to the ICH guidelines revealed that the method is selective and stability-indicating. The proposed method is simple, accurate, precise, and specific, and it has the ability to separate the drug from degradation products and excipients found in the dosage form. The method is suitable for the routine analysis of DUL in tablets. In addition, the HPLC procedure can be applied to the analysis of samples obtained during accelerated stability experiments to predict expiration dates of pharmaceuticals.

## Experimental

### Apparatus

A Series 200 HPLC system (PerkinElmer, Shelton, CT) equipped with a Series 200 diode array detector, Series 200 quaternary gradient pump, Series 200 column oven, manual injector rheodyne valve) with 20 μL fixed loop, Turbochrom navigator software (Version 6.1.1.0.0:K20), and Hypersil C18 column (150mm× 4.6mmid, 5 μm particle size) was used.

### Reagents and Materials

○ Pure samples: Analytically pure powder Duloxetine hydrochloride was procured as gratis samples from Sun Pharmaceuticals Limited, Gujarat, India.
○ Chemicals and reagents: HPLC grade water, acetonitrile and orthophosphoric acid was purchased from E. Merck (Mumbai, India).
○ Market samples: Tablets containing Duloxetine HCl (20mg) of brand Torrent Pharmaceuticals Ltd., Ahmedabad (Gujarat, India) were purchased from the local market.

### Chromatographic Conditions

The Hypersil C_18_ column (18) was used at ambient temperature. The mobile phase consisted of acetonitrile-buffer (0.01 M Potassium dihydrogen phosphate, pH 5.4 adjusted with ortho-phosphoric acid) (50:50, v/v) and the flow rate was maintained at 1 ml/min. The mobile phase was passed through nylon 0.45 μm–47 mm membrane filter and degassed before use. The elution was monitored with UV detector at 229 nm, and the injection volume was 20 μL.

HPLC method depends upon the nature of the sample (ionic or ionizable or neutral molecule), its molecular weight and solubility. UPLC was selected for the initial separation because of its simplicity and suitability. To optimize the chromatographic conditions the effect of chromatographic variables such as mobile phase, pH, flow rate and solvent ratio were studied. The resulting chromatograms were recorded and the chromatographic parameters such as capacity factor, asymmetric factor, and resolution and column efficiency were calculated. The condition that gave the best resolution, symmetry and capacity factor was selected for estimation.

### Preparation of DUL Standard Stock Solutions (100μg/ml)

Accurately weighed 25 mg of DUL transferred to a 25ml volumetric flask and dissolved and diluted to the mark with methanol to obtain a standard solution of 1000 μg/ml. This solution (1 ml) was further diluted to 10 ml with mobile phase to obtain a working standard stock solution of 100μg/ml for the RP- HPLC method.

### Preparation of Sample Solutions

Twenty tablets were weighed and finely powdered. A mass equivalent to 20 mg of DUL was weighed and transferred in a 100 ml volumetric flask, mixed with methanol (60 ml), and sonicated for 20 min. The solution was filtered through Whatman filter paper No. 41, and the residue was washed thoroughly with methanol. The filtrate and washings were combined in a 100 ml volumetric flask and diluted to the mark with methanol. An aliquot of this solution (0.2 ml) was further diluted to 10 ml with methanol to obtain a solution containing 4 μg/ml of DUL and subjected to RP-HPLC analysis.

### Method Validation

#### Linearity and range

Calibration curves were constructed by plotting peak areas versus concentrations of DUL, and the regression equations were calculated. The calibration curves were plotted over the concentration range 1–25 μg/ml. Accurately measured standard working solutions of DUL (0.1, 0.2, 0.4, 0.8, 1.0, 2.0, and 2.5ml) were transferred to a series of 10 ml volumetric flasks and diluted to the mark with mobile phase. Aliquots (20 μL) of each solution were injected under the operating chromatographic conditions described above.

#### Accuracy (recovery)

The accuracy of the method was determined by calculating recoveries of DUL by the standard addition method. Known amounts of standard solutions of DUL (50, 100, and 150%) were added to prequantified sample solutions of tablets. The amounts of DUL were determined by applying these values to the regression equation of the calibration curve.

#### Method precision (repeatability)

The precision of the instruments was checked by repeatedly injecting (n = 6) solutions of DUL (5 μg/ml) for the RP-HPLC method.

#### Intermediate precision

Precision was evaluated in terms of intraday and interday precision. The intraday precision was investigated using different concentrations (1, 5, 25 μg/ml) of standard solutions and sample solutions. The intraday and interday precisions of the proposed methods were determined by estimating the corresponding responses three times on the same day and on three different days over a period of 1 week for different concentrations of DUL standard and sample, respectively. The results were reported in terms of RSD.

#### Robustness

To determine the robustness of the developed method, experimental conditions were deliberately altered and the effect on resolution was recorded. There was no detrimental effect on the method performance as shown. Low value of relative standard deviation was indicating that the method was robust.

#### LOD and LOQ

The LOD was determined by the analysis of samples with known concentrations of analyte and by establishing through visual evaluation the minimum level at which the analyte could be reliably detected. The LOQ was determined by the analysis of samples with known concentrations of analyte and by establishing the minimum level at which the analyte could be quantified with acceptable accuracy and precision.

#### Specificity

To assess the method specificity, tablet powder without DUL was prepared with the same excipients as those of in the commercial formulation. For RP-HPLC, the solution was prepared using the same procedure as for the analytical sample. Moreover, to evaluate the influence of the putative degradation products on the resolution of DUL, a standard stock solution was prepared as reported above, except for the addition of DUL at a 1 μg/ml concentration. HPLC analysis was performed after dilution, as reported for the working standard solution. Resolution factors were calculated with the LC solution software. The specificity of the method was established through study of resolution factors of the drug peak from the nearest resolved peak, and among all other peaks. Selectivity was confirmed through peak purity studies using the UV detector.

#### System suitability

A system suitability test was an integral part of the method development to verify that the system is adequate for the analysis of DUL to be performed. The suitability of the chromatographic system was demonstrated by comparing the obtained parameter values with the acceptance criteria of the U.S. Food and Drug Administration, Center for Drug Evaluation and Research guidance document (U.S. Food and Drug Administration, 1994). A system suitability test of the chromatography system was performed before each validation run. Six replicate injections of a system suitability/calibration standard and one injection of a check standard were made. Area, retention time (RT), tailing factor, asymmetry factor, and theoretical plates for the six suitability injections were determined.

#### Stability of standard and sample solutions

**Stability of standard and sample solution of DUL was evaluated at room temperature for 48 hr. The relative standard deviation was found below 2.0%. It showed that both standard and sample solution were stable up to 48 hr at room temperature.**

### Determination of DUL in Tablets

Tablets of DUL were purchased from a local market. The responses of tablet solutions measured with the UV detector showed a wavelength maximum at 229 nm for the RP-HPLC method. The amounts of DUL present in sample solution were determined by fitting the responses into the regression equation for DUL.

### Forced Degradation of DUL

DUL is practically insoluble in water but very soluble in methanol; therefore, methanol was used as the solvent in all studies. After forced degradation studies all the resultant solutions were diluted using methanol to obtain final concentration of 10 μg/ml of DUL.

- Oxidation: Solutions of DUL (10 μg/ml) for oxidation studies were prepared using 3% H_2_O_2_ in methanol, and the resultant solutions were stand for 24 hr to facilitate oxidation of the DUL.
- Acid degradation: Solutions of DUL (10 μg/ml) for acid degradation studies were prepared using 0.1 M HCl in methanol and the resultant solutions were stand for 24 hr.
- Alkali degradation: Solutions of DUL (10 μg/ml) for alkali degradation studies were prepared using 0.1 M sodium hydroxide in methanol, and the resultant solutions were stand for 24 hr.
- Dry heat: For dry heat degradation studies drug powder was exposed in an oven (80 °C) for 2 days. The solids were allowed to cool. DUL was accurately weighed and transferred to a volumetric flask containing few ml of methanol. The volume was made up to the mark with methanol. The solution was further diluted with methanol to obtain final concentration of 10 μg/ml of DUL.
- Photolytic degradation: For photolytic degradation study drug powder was exposed to direct sunlight for 24 hr. After exposure, solid was accurately weighed and transferred to volumetric flask containing few ml of methanol. The volume was made up to the mark with methanol. The solution was further diluted with methanol to obtain final concentration of 10 μg/ml of DUL (ICH, Q1B, 1996).

## Figures and Tables

**Tab. 1.** System suitability test parameters for Duloxetine hydrochloride at the proposed HPLC method

**Parameter**	**Duloxetine HCl**
Retention times (R_T_)	5.84 Min
HPLC Plate Count	6394
Tailing factor	1.27
Base width (sec)	20.98

**Tab. 2.** Regression analysis of calibration graphs for Duloxetine hydrochloride by proposed HPLC method

**Parameter**	**Duloxetine hydrochloride**
Linearity (μg/ml)	1–25
Correlation co –efficient (r)	0.998
Slope of Regression(S)	80042
Intercept of Regression	72864
Standard deviation of slope	23.1
Standard deviation of intercept	63.53

**Tab. 3.** Data derived from accuracy of Duloxetine hydrochloride the proposed HPLC method

**Amount of Sample (μg/ml)**	**Sets**	**Amount drug of spiked (μg/ml)**	**Area(n=3)**	**Average amount recovered (μg/ml)**	**% Recovery**	**Mean % Recovery**	**% RSD^a^**
4	1	0	244245.3		99.04		
	2	0	246259.7	3.99	99.67	99.78	0.84
	3	0	248358.1		100.32		

4	1	2	410442.1		100.9		
	2	2	407248.4	6.02	99.96	100.68	0.51
	3	2	411122.8		101.17		

4	1	4	569276.0		100.56		
	2	4	571411.2	8.03	101.2	100.71	0.25
	3	4	568677.9		100.38		

4	1	6	732162.2		101.44		
	2	6	731173.0	10.05	101.13	101.21	0.089
	3	6	730944.2		101.06		

a RSD = Relative standard deviation.

**Tab. 4.** Summary of validation parameters for Duloxetine hydrochloride the proposed HPLC method

**Parameters**	**Duloxetine hydrochloride**
LOD (μg/ml)^a^	0.0257
LOQ (μg/ml)^b^ n=5	0.0779
Accuracy, %	99.8–101.3
Repeatability, (% RSD, *n* = 6)	0.0050–0.303
Precision (% RSD)	
Interday (n = 3)	0.11–0.56
Intraday (n = 3)	0.12–0.36

a LOD = Limit of detection;

b LOQ = Limit of quantitation.

**Tab. 5.** Data derived from robustness of Duloxetine hydrochloride the proposed HPLC method

**Parameters**	**Normal condition**	**Change in condition**	**Change in % RSD**
Flow Rate	1.0 ml/min	0.9 ml/min	0.11
		1.1 ml/min	0.25
pH	5.4	4.9	0.083
		5.9	0.11
Mobile phase ratio	50:50	45:55	0.079
		55:45	0.17

**Tab. 6.** Assay results for Duloxetine hydrochloride in marketed tablet dosage form by proposed HPLC method

**Tablet**	**Concentration (μg/ml)**	**Amount recovered (μg/ml)**	**Duloxetine HCl ± SD*a* (*n* =3), %**
A	4	3.99	99.75 ± 2.056

a SD = Standard deviation

**Tab. 7.** Forced degradation study for Duloxetine HCl the proposed HPLC method

**Condition**	**Time (h)**	**% Recovery DUL**	**Retention time of degradation products**
Base 0.1 N NaOH	24	99.9	–
Acid 0.1 N HCl	24	2.86	2.49, 3.04, 3.77 and 7.21
3% hydrogen peroxide	24	100.7	–
Dry heat^a^	24	99.7	–
Sunlight	24	99.3	–

a Samples were heated at 80 ° for specified period of time.
